# Evaluation of a high-throughput, cost-effective Illumina library preparation kit

**DOI:** 10.1038/s41598-021-94911-0

**Published:** 2021-08-05

**Authors:** Eric S. Tvedte, Jane Michalski, Shaoji Cheng, Rayanna S. Patkus, Luke J. Tallon, Lisa Sadzewicz, Vincent M. Bruno, Joana C. Silva, David A. Rasko, Julie C. Dunning Hotopp

**Affiliations:** 1 Institute for Genome Sciences, University of Maryland School of Medicine, Baltimore, MD 21201 USA; 2 Department of Microbiology and Immunology, University of Maryland School of Medicine, Baltimore, MD 21201 USA; 3 Department of Medicine, University of Pittsburgh, Pittsburgh, PA 15261 USA; 4 Greenebaum Cancer Center, University of Maryland School of Medicine, Baltimore, MD 21201 USA

**Keywords:** Genomics, Pathogens

## Abstract

Library preparation for high-throughput sequencing applications is a critical step in producing representative, unbiased sequencing data. The iGenomX Riptide High Throughput Rapid Library Prep Kit purports to provide high-quality sequencing data with lower costs compared to other Illumina library kits. To test these claims, we compared sequence data quality of Riptide libraries to libraries constructed with KAPA Hyper and NEBNext Ultra. Across several single-source genome samples, mapping performance and de novo assembly of Riptide libraries were similar to conventional libraries prepared with the same DNA. Poor performance of some libraries resulted in low sequencing depth. In particular, degraded DNA samples may be challenging to sequence with Riptide. There was little cross-well plate contamination with the overwhelming majority of reads belong to the proper source genomes. The sequencing of metagenome samples using different Riptide primer sets resulted in variable taxonomic assignment of reads. Increased adoption of the Riptide kit will decrease library preparation costs. However, this method might not be suitable for degraded DNA.

## Introduction

Despite the decreased costs and increased throughput of the latest sequencing technologies, library preparation remains a labor-intensive and expensive bottleneck. For small genomes, like bacterial genomes, which can be extensively multiplexed, library construction can be the most expensive laboratory step. Therefore, efforts are needed to reduce labor and reagent costs in library construction while generating high-quality whole genome sequencing data.

There are hundreds of modern library construction methods. While methods vary with respect to input DNA requirements, reagent costs, and mechanism of action, paramount is the objective to generate an unbiased library from the source DNA^1^. For Illumina-based sequencing, standard kits typically require nanograms to micrograms of input DNA, although workflows incorporating picogram quantities have been reported^2,3^. Methods using PCR amplification steps enable sequencing of samples with low input DNA but have been associated with systematic bias (e.g. underrepresentation of GC and AT rich regions)^4–6^. Biases can be attributed to genome content^7,8^, input DNA quality^9^, and sequencing platform^10^.

The iGenomX Riptide High Throughput Rapid Library Prep Kit (https://igenomx.com/products/) promotes ultrafast and automatable processing of biological samples to yield high-quality Illumina sequence data. Libraries are constructed by polymerase-mediated extension of barcoded random primers, circumventing several time- and reagent-consuming steps in genomic library preps, including DNA fragmentation, end repair, and ligation. The use of sample barcodes allows simultaneous processing of up to 96 samples for fragment capture, second strand synthesis, PCR amplification, and Illumina sequencing. The marketed price of a Riptide kit represents a major reduction in library costs to < $10 per sample.

Given the potential to reduce library preparation costs for labs performing high-volume genome sequencing experiments, we sought to independently test Riptide libraries for diverse organisms that are the causative agents of multiple infectious diseases. One primary application of Riptide is whole genome shotgun (WGS) sequencing of microbial samples, and we conducted multiple assessments of sequencing data quality in single-source prokaryotic samples in addition to metagenomes. Riptide libraries were compared to widely-used Illumina libraries (KAPA Hyper, NEBNext Ultra) generated from the same DNA sample. While Riptide was not marketed toward WGS of eukaryotic samples, we also performed sequencing experiments to investigate whether multiple Riptide libraries could be sequenced and merged as an alternative to conventionally prepared libraries.

## Results

### Methods overview

Riptide libraries were prepared on two 96-well plates. On the first plate, Riptide libraries were constructed from genomic DNA from ten biological specimens (Table 1; Supplementary Data S1) including prokaryotic, eukaryotic, and plasmid samples. Technical replicates (3–32 replicates per species) were performed to assess library variability in prokaryotic samples and merge libraries in eukaryotic samples. The samples included degraded gDNA from *Shigella boydii* and *Cryptosporidium parvum* as well as blank wells containing no input gDNA. Libraries were prepared with recommended primer sets based on organismal GC content.

**Table 1:** Biological samples used in the study.

Specimen	Strain	Riptide replicates	Assembly size (bp)	% GC	Genbank assembly accession
*Acinetobacter baumannii*	25493-3	4	4,073,396	39.5	GCA_000600795.1
*Aspergillus fumigatus*	925	8	29,384,958^a^	49.8	GCA_000002655.1^‡^
*Aspergillus fumigatus*	928	8	29,384,958^a^	49.8	GCA_000002655.1^‡^
*Aspergillus fumigatus*	H7810724	12	29,384,958^a^	49.8	GCA_000002655.1^‡^
*Aspergillus fumigatus*	T8015994	4	29,384,958^a^	49.8	GCA_000002655.1^‡^
*Brugia pahangi* + *Wolbachia*	FR3	10	98,070,936	28.0	GCA_012070555.1 NZ_CP050521.1
*Cryptosporidium parvum*	TU114	3	9,106,213^a^	30.2	GCA_003148445.1
*Escherichia coli*	HS	10	4,643,538	50.8	GCA_000017765.1
*Klebsiella pneumoniae*	4006	4	5,563,933	57.3	GCA_000699405.1
pB171	*E. coli* B171	4	159,043	50.9	CP021211.1 CP021212.1
*Plasmodium falciparum*	NF54	6	23,452,658	19.4	N/A
*Shigella boydii*	5216-82	8	4,882,454	50.8	GCA_000211975.2
*Theileria parva*	Muguga	4	8,347,606	34.0	GCA_000165365.1

^a^Assembly size includes gap regions. ^‡^*A. fumigatus* Af293 strain.

On the second Riptide plate, libraries were generated for four enterotoxigenic *E. coli* (ETEC) metagenome samples (Supplementary Data S1). Metagenome libraries were prepared using multiple sets of primers to assess how primer selection effects metagenome composition, and two technical replicates were sequenced for each sample-primer combination. The second Riptide plate also included libraries for four additional strains of *Acinetobacter baumannii*, which had an unexpected low yield of reads sequenced on the first Riptide plate.

Sequencing throughput and contamination were assessed for all Riptide libraries. To conduct additional tests of Riptide sequencing data quality, the Riptide sequencing data was compared to sequencing data from KAPA Hyper libraries from two prokaryotic samples (*E. coli*, *Klebsiella*) that were generated after Riptide sequencing and from two eukaryotic samples (*Aspergillus*, *Brugia*) that were generated prior to Riptide sequencing (Supplementary Data S1).

### Read representation in Riptide samples

**Figure 1:**
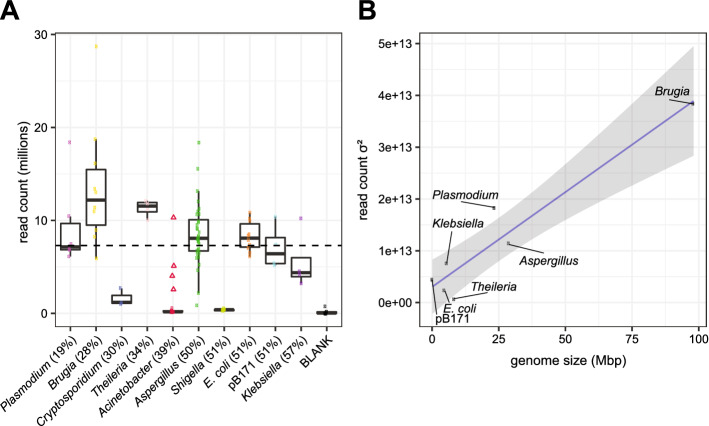
Riptide library read composition across study specimens. (**A**) Variability of sequencing throughput was assessed by examining the distribution of read counts per taxa. Members of a read pair were counted as two reads. Boxplots were drawn without outliers, and values from individual replicates are overlaid as colored dots. The red triangles shown without a corresponding boxplot denote four different *Acinetobacter* strains sequenced on a second Riptide plate (Supplementary Data S1). Specimens are organized along the x-axis by increasing genome GC content, which are shown in parentheses. The dashed horizontal line displays the expected 7.3 million read count per Riptide library by dividing the total sequencing throughput (654 million) by the number of Riptide libraries containing gDNA (89). (**B**) For each of seven species having consistent amplification of Riptide libraries, the variance (σ^2^) of read counts was calculated assuming values represent entire populations. Genome size was plotted against variance, and a linear regression with standard error was estimated using ggplot2 in R.

The sequencing run of the first Riptide plate produced ~ 654 million reads (~ 327 read pairs) meaning the 89 libraries containing gDNA averaged 7.3 million reads. Sequencing of libraries was successful for seven of ten biological samples including prokaryotes and eukaryotes (Fig. 1A). Low read counts were observed for *Shigella* and *Cryptosporidium*, which had low-quality input DNA (Supplementary Fig. S1); although Illumina reads were subsequently successfully generated from KAPA libraries constructed from the same *Shigella* gDNA (Supplementary Data S1). Low reads in the *Acinetobacter samples* were likely a technical error as other *Acinetobacter* strains were sequenced successfully on a later Riptide plate, although we cannot rule out that the Ab25493-3 DNA on the first Riptide plate inhibited DNA amplification (Fig. 1A). Prokaryotic and small eukaryotic genomes libraries had more consistent read yields (Fig. 1B) with quick and efficient library pooling.

### Contamination analysis of Riptide samples

**Figure 2:**
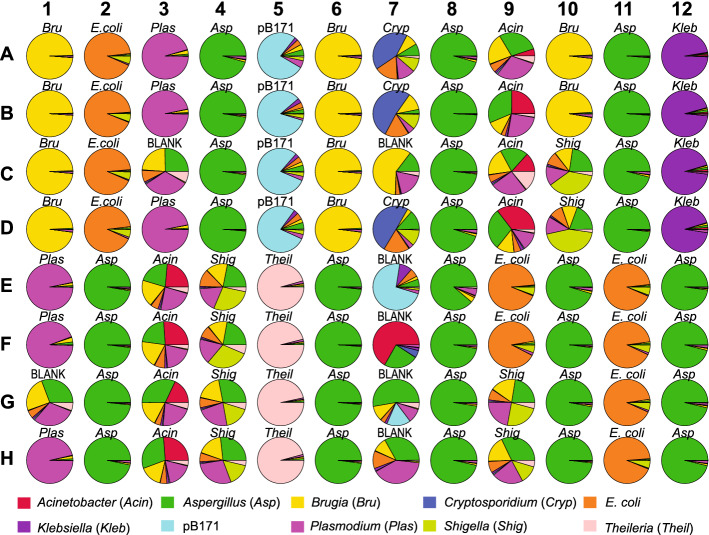
Contamination assessment of Riptide samples. To characterize contamination in Riptide libraries, primary reads were mapped to a reference containing genomes from all specimens used in the study. Pie charts display proportions of reads mapping to each genome. The figure is organized to represent the actual position of samples loaded on the Riptide 96-well plate. Wells are labeled according to source DNA used to prepare the Riptide libraries; wells containing no gDNA are labeled as BLANK.

To assess potential contamination across Riptide samples in the 96-well plate, reads were mapped with bwa mem^11^ to a combined reference sequence that contained all genomes used in this study. Excluding samples from degraded DNA and negative controls, 64/70 Riptide libraries had > 90% reads mapped to the proper source genome (i.e. < 10% of reads mapped to other genomes) and 51/70 had > 95% reads properly mapped (Fig. 2). One potential source of erroneous short read mapping is microhomology between species. This was most apparent in *E. coli* samples which had ~ 5% reads mapping to *Shigella*. Alignments to the large *Aspergillus*, *Brugia*, and *Plasmodium* genomes were also common. When low quality mappings with a MAPQ score < 20 were removed from the dataset using SAMtools^12^, 66/70 Riptide libraries had > 90% reads matching the proper source genome and 63/70 had > 95% properly mapped reads (Supplementary Fig. S2).

Seven negative control Riptide libraries containing no source gDNA were prepared and sequenced (Fig. 2). One negative control library prepared next to four *Brugia* libraries (well C7) had 358,451 reads mapped to *Brugia*. Two negative controls (wells G1 and C3) were close to *Aspergillus*, *Brugia*, and *Plasmodium* libraries and had > 25,000 reads mapping to each of these species. The remaining four negative controls had fewer than 30,000 total reads and had more variable mapping patterns. Libraries prepared from degraded DNA also had reads corresponding to multiple genomes; the eight *Shigella* libraries and three *Cryptosporidium* libraries had > 50% of reads mapping to other species. Sequencing errors altering the sample barcode sequence could lead to misassignment. The 96 Riptide libraries sequenced during a single run had a shared plate barcode sequence, therefore the sample barcode was the only potential source of misassignment. A given Riptide sample barcode would require at least two specific nucleotide changes to be improperly assigned; all pairwise comparisons of barcodes (4560/4560) had a pairwise distance value > 1. Nearly all barcodes would require at least three nucleotide changes, with over 98% (4474/4560) of pairwise comparisons having a distance value > 2. Thus, the incidence of misassignment due to sequencing errors is presumed to be minimal. Another common source of contamination in Illumina sequencing is the nonspecific binding of free adapters when sequencing is performed on multiplexed libraries, which is known as index hopping^13,14^. Free adaptors are excluded during streptavidin bead capture therefore index hopping should not be prevalent in Riptide libraries. Since the control wells had a composition that more closely matched that of adjacent wells, as opposed to the entire plate, it seems that if index hopping happens it is less frequent than well-to-well DNA contamination, at least in this experiment.

### Read mapping performance in Riptide samples

Two prokaryote (*E. coli*, *Klebsiella*) and two eukaryote (*Aspergillus*, *Brugia*) samples provided the most robust comparisons of read mapping with Riptide data. These Riptide libraries contained ample read data with minimal contamination and had KAPA Hyper libraries prepared on the same gDNA. We did not attempt to sequence at the same depth in Riptide and KAPA libraries, therefore we randomly downsampled to a common sequencing depth before mapping.

**Figure 3:**
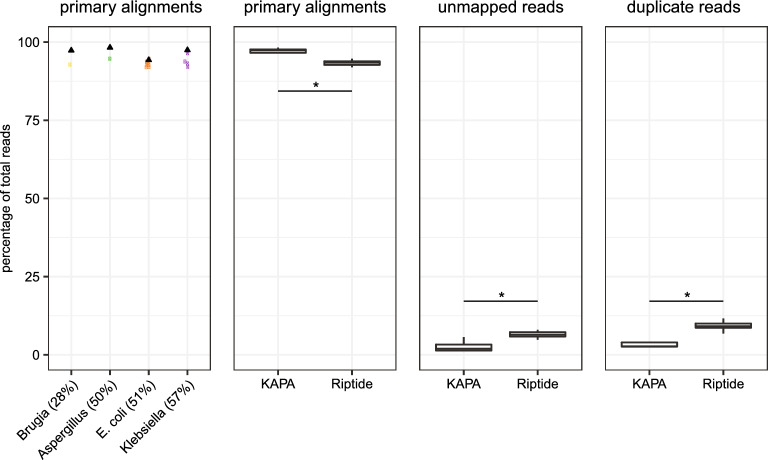
Comparison of mapping performance in Riptide versus KAPA Hyper libraries. (**A**) The percentage of primary alignments in Riptide and KAPA Hyper libraries were determined after mapping randomly subsampled read sets to individual genomes and subsequent removal of unmapped reads, duplicate reads, secondary alignments (repeats), and supplementary alignments (chimeras). secondary alignments. SAMtools was used to filter read sets. Colored circles denote Riptide libraries, black triangles denote KAPA Hyper libraries. After grouping all Riptide libraries (n = 16) and all KAPA libraries (n = 4), a Welch’s t test was performed to compare the percentages of (**B**) primary alignments, (**C**) unmapped reads, and (**D**) duplicated reads. *Differences between Riptide and KAPA Hyper distributions were significant (p value < 0.05).

Reads were mapped to the respective reference genome using bwa mem, and mapping performance statistics were produced using Picard (https://broadinstitute.github.io/picard/). Riptide libraries had > 91% of total reads mapped as primary alignments, and percentages were consistent across *E. coli* and *Klebsiella* replicates (Fig. 3). There was no discernible GC bias in the proportion of mapped reads. The four KAPA libraries tended to have a higher percentage of primary alignments relative to the 16 Riptide datasets (p = 0.02) (Fig. 3). Percentages of unmapped and duplicated reads were significantly higher in Riptide libraries (p = 0.03 and p = 0.01, respectively) (Fig. 3).

**Figure 4:**
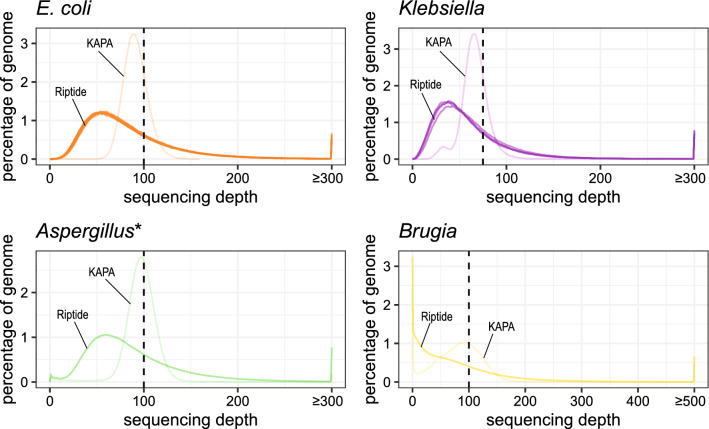
Histograms of sequencing depth in Riptide and non-Riptide libraries. For each library, randomly subsampled reads were mapped to a reference containing the source genome, and non-primary alignments were subsequently removed. All Riptide replicates are shown for *E. coli* and *Klebsiella*, while distributions for *Aspergillus* and *Brugia* were generated from merged Riptide libraries. Vertical dotted lines correspond to the expected mode of distributions based on the sequencing depth targeted with subsampling procedures. *Regions having zero sequencing depth which largely represent tracts of Ns in the scaffolded *Aspergillus* assembly were omitted from this plot.

Riptide libraries had more asymmetric distributions of sequencing depth relative to KAPA libraries, and the modes of KAPA distributions were closer to expected values (Fig. 4; Supplementary Fig. S3). The long right tails of *E. coli*, *Klebsiella*, and *Aspergillus* distributions indicate the overrepresentation of certain regions of the genome in Riptide sequencing data. There was no apparent local maxima in the *Brugia* Riptide distribution despite merging replicates, but may be reflective of its large genome size (100 Mbp; Fig. 4). While both Riptide and KAPA libraries had similar amounts of the genome sequenced up to the targeted sequencing depth, the tighter distribution around the targeted depth in KAPA libraries is reflected in cumulative and non-cumulative frequency distributions (Fig. 4; Supplementary Fig. S3).

In *E. coli* and *Klebsiella*, both Riptide and KAPA libraries had evidence of GC bias, as assessed with Picard by aggregating on a sliding window (Supplementary Fig. S4). In *E. coli*, regions with the GC content similar to the genome-wide average (~ 50%) tended to have normalized sequencing depth close to one. Regions with < 50% GC were overrepresented in sequencing data, and regions with > 50% were underrepresented. A similar pattern was observed in *Klebsiella* with a shifted distribution reflecting the higher GC content of this bacteria (~ 57%) Supplementary Fig. S4). GC bias was less apparent in *Aspergillus* libraries, which had more even sequencing depth across spectrum of GC content (Supplementary Fig. S4). In *Brugia,* KAPA libraries had normalized sequencing depth close to one across genome regions containing 15–50% GC, but there was a bias in *Brugia* Riptide libraries as 15–25% GC regions were underrepresented and 30–50% GC regions were overrepresented (Supplementary Fig. S4). This result suggests the abundance of low sequencing depth regions in *Brugia* is at least partially attributable to GC bias in Riptide sequencing.

### De novo assembly evaluation

Subsampled read sets were assembled using SPAdes^15^ and contigs > 1 kbp were evaluated using QUAST^16^ and BUSCO^17,18^*.* Bacterial assemblies generated with Riptide libraries contained more contigs and had lower contig N50 values relative to KAPA assemblies (Table 2). Bacterial assembly size was similar across Riptide replicates and KAPA, with over 97% of the reference *E. coli* genome and over 96% of the reference *Klebsiella* genome aligned to assembly contigs regardless of library preparation (Table 2; Supplementary Data S1). Of the 148 bacterial BUSCO genes, 146 were identified in all *E. coli* Riptide replicates and 143–145 were identified in *Klebsiella* replicates (Table 2; Supplementary Data S1). Over 96% of previously annotated *E. coli* genes and 94% of annotated *Klebsiella* genes were found in Riptide assemblies using QUAST (Supplementary Data S1).

**Table 2:** Quantitative summaries of de novo assemblies.

Specimen	Library	Assembly size (Mbp)	Contigs	Contig N50 (bp)	Largest contig (bp)	Mismatch rate^a^	Indel rate^a^	BUSCO complete^b^
*E. coli*	Riptide	4.57–4.71	110–199	74,933–83,433	187,066–266,682	1.94–3.15	0.2–0.48	146/148
*E. coli*	KAPA	4.64	90	85,968	247,378	2.18	0.18	146/148
*Klebsiella*	Riptide	5.45–5.56	110–186	88,948–105,043	246,750–329,070	3.15–3.61	0.26–0.45	143/148–145/148
*Klebsiella*	KAPA	5.45	75	130,769	521,945	4.25	0.46	145/148
*Aspergillus*	Riptide	29.12	1454	91,853	303,741	162.96	18.64	296/303
*Aspergillus*	KAPA	28.08	405	177,655	682,945	172.13	18.79	297/303
*Brugia*	Riptide	77.48	23,822	4330	84,648	159.81	52.05	189/303
*Brugia*	KAPA	80.68	6903	25,762	262,303	146.35	59.72	277/303

All statistics are shown for contigs > 1 kbp. Full results are available in Supplementary Data S1. ^a^Mismatch rate and indel rates were calculated as the number of mismatches/indels in the assembled contigs relative to the reference genome per 100 kbp of aligned sequence. ^c^BUSCO complete is the number of identified single copy + duplicated BUSCOs.

Assemblies of eukaryotic genomes using Riptide sequencing were also less contiguous relative to KAPA, but the differences were more pronounced with contig N50 values ~ 2-fold higher in the *Aspergillus* KAPA library and ~ 5-fold higher in the *Brugia* KAPA library (Table 2). The *Aspergillus* Riptide assembly included 97% of eukaryotic BUSCO genes and 96% of annotated *Aspergillus* genes, similar to observations in the KAPA assembly (Table 2; Supplementary Data S1). In *Brugia*, the Riptide contigs aligned to 9% less of the reference *Brugia* genome and contained 29% fewer eukaryotic BUSCO genes when compared to the KAPA assembly (Table 2; Supplementary Data S1). Given the lower overlap between the Riptide contigs and *Brugia* reference genome, either the BUSCO gene regions are not represented in the *Brugia* Riptide libraries or they are located in highly fragmented portions of the assembly (contigs < 1 kbp).

To estimate assembly per-base accuracy, mismatch rates (MMR) and indel rates (IDR) were calculated as the number of mismatches and indels, respectively, per 100 kbp aligned sequence between assembled contigs and reference genomes. Bacterial assemblies were > 99.99% identical, having ≤ 4.25 MMR and ≤ 0.48 IDR across all *E. coli* and *Klebsiella* replicates (Table 2). Eukaryotic assemblies were ~ 99.8% identical, with both MMR and IDR tens- to hundreds-fold higher in *Aspergillus* and *Brugia* relative to rates in prokaryotes (Table 2). There was no consistent relationship between percent identity and Illumina library preparation method. The *E. coli* KAPA assembly MMR fell within the range of Riptide replicates whereas the IDR was lower. Both MMR and IDR were higher in the *Klebsiella* KAPA assembly relative to Riptide replicates. The *Brugia* KAPA assembly had a lower MMR and higher IDR than Riptide, while both measures were lowest in the *Aspergillus* Riptide assembly. Overall, percent identity was similar across assemblies produced with different library methods.

### Comparison of metagenome sequencing across library preparation methods

There are three primer choices for library construction with Riptide sequencing: (a) high GC, (b) low GC, and (c) a 1:1 mixture of high:low GC. To examine if Riptide libraries could be used to sequence metagenome libraries and how primer choice influences sample composition, libraries were produced from stool metagenomic samples acquired from an enterotoxigenic *E. coli* (ETEC) infected individual (day 1) and stools from three additional experimental time points (day 2, day 6, day 10)^19^. The infected individual was treated with antibiotics on day 6 and resolved the diarrhea by day 10. NEBNext Ultra libraries were prepared from the same source DNA to provide comparisons to Riptide libraries.

**Figure 5:**
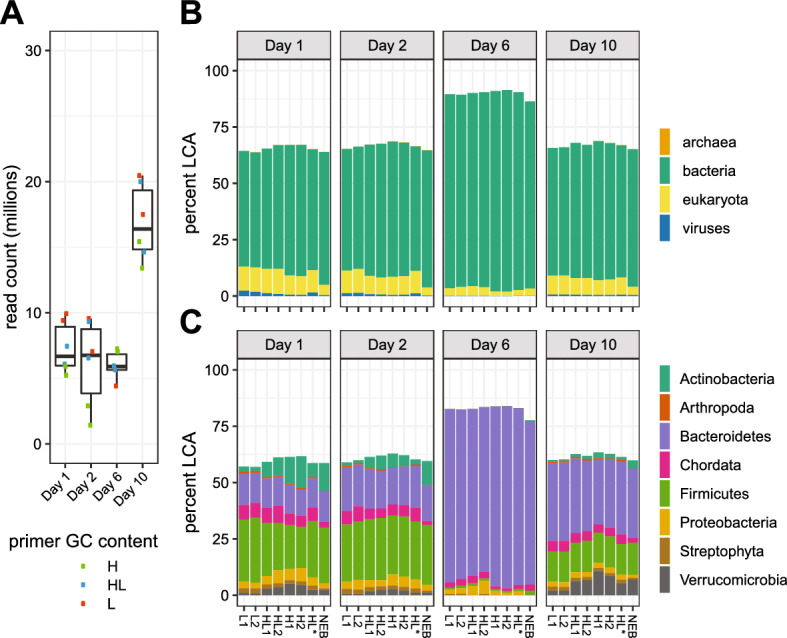
Read composition and taxonomic assignment of ETEC metagenome libraries. (**A**) Raw read counts of Riptide libraries using metagenomic samples. Variability of sequencing throughput was assessed by examining the distribution of read counts per metagenome sample. Members of a read pair were counted as two reads. Circles denote individual values. Libraries are grouped along the x-axis by sample source. The colors of circles correspond to GC primer used in library preparation: L = low GC, HL = 1:1 high:low GC, H = high GC. (**B**,**C**) Taxonomic classification of reads from ETEC metagenomic samples. Classification of reads from ETEC metagenomic samples was performed using kraken2 with NCBI NT as the reference database. Samples are organized along the x-axis by GC primer used in library preparation, L = low GC, HL = 1:1 high:low GC, H = high GC. Mock high:low datasets were constructed by combining high and low GC datasets for each sample (HL*). NEBNext Ultra libraries were also prepared for each sample (NEB). The lowest common ancestor (LCA) was determined for each read in the dataset, after which percentages of reads corresponding to various taxonomic ranks were obtained. (**B**) Reads were classified at the Domain rank. (**C**) Reads were classified at the Phylum rank, filtered to include those with > 0.5% total reads assigned.

All Riptide metagenome libraries had > 1 million reads sequenced, and read counts were primarily associated with experimental time points (Fig. 5A, Supplementary Data S1). Samples from days 1, 2, and 6 had overlapping read count distributions, with median values of 6.7, 6.7, and 5.9 million reads, respectively. Read counts for day 10 samples were much higher, with a median value of 16.4 million. NEBNext libraries had higher read counts relative to Riptide (day 1: 84.0 million, day 2: 96.7 million, day 6: 81.9 million, day 10: 57.7 million), but this was expected as there was no attempt to target the same depth with the two library methods.

Assignment of reads to domain and phylum taxonomic ranks was performed using kraken2^20^. Total percentages of classified reads were consistent, ranging from 69 to 75% in days 1, 2, and 10, and 95 to 97% in day 6 samples. Percentages of assigned reads in NEBNext libraries were similar to Riptide samples (day 1: ~ 70%, day 2: ~ 71%, day 6: ~ 95%, day 10: ~ 71%). At the domain rank, Bacteria was the prominent taxonomic assignment (Fig. 5B). In day 1, day 2, and day 10 samples, higher primer GC content is associated with more Bacteria assignments and fewer Eukaryota assignments. Assignment to viruses also decreased with higher primer GC in day 1 and day 2 samples.

Phylum assignments were similar in Riptide libraries from sample days 1, 2, and 10. (Fig. 5C). In these samples, a larger proportion of reads were assigned to Actinobacteria and Verrucomicrobia as the GC content of library primers increased, which are both high GC^21,22^. There was no consistent pattern in taxonomic assignment and primer GC in Bacteroidetes, Firmicutes, or Proteobacteria.

Combining readsets from high and low primer GC (HL* in Fig. 5B,C) produced taxonomic profiles similar to samples sequenced with both primers (HL). NEBNext libraries produced from metagenomes showed similar taxonomic profiles to their Riptide counterparts (Fig. 5B,C). Notably, the ratio of bacteria to eukaryote assignments was higher in NEBNext libraries relative to Riptide libraries in day 1, 2, and 10 samples, and Chordata assignments were lowest in these libraries.

Day 6 samples had a higher number of classified reads and a distinct assignment pattern likely associated with severe ETEC infection occurring at this specific time point. Proteobacteria was underrepresented and Firmicutes and Actinobacteria were overrepresented in the NEBNext library relative to multiple Riptide libraries (Fig. 5B,C). Differences in representation across libraries were small and are obscured by the overwhelming proportion of *Bacteriodetes* spp*.* at this experimental time point. Overall, there was no obvious systematic pattern across all samples, and most assignments in the NEBNext libraries fell within the range of the Riptide replicates.

## Discussion

Multiple assessments of data quality support the use of the Riptide kit as a viable option for NGS library preparation. Single Riptide libraries provide ample read data for prokaryotes or small eukaryotic genomes, while merging datasets can achieve desired sequencing depth for large eukaryotic genomes. Despite its potential to produce data suitable for a wide range of short read sequencing applications, conventional Illumina library preparations should be the preferred option in certain situations like those with degraded DNA samples, which can frequently occur with difficult to obtain samples. While we did not explicitly measure the effects of different gDNA impurities, we tested a diverse set of gDNA samples prepared by multiple individuals with a variety of different methods under real laboratory conditions. Further studies could clarify how varying concentrations of common gDNA contaminants such as guanidinium ions, phenol/chloroform traces, protein, and salts could hinder library amplification.

While the observed sequencing depth in Riptide libraries approximated expected values, distributions often had asymmetrical distributions with long right tails. It has been previously demonstrated that Illumina libraries have biased sequencing depth associated with GC content^9,23^. In the diverse set of biological samples surveyed here, we observed examples of relatively uniform sequencing depth across variable GC genomic regions in addition to examples of GC bias. In most cases, GC bias was similar in Riptide and KAPA libraries. However, there was a notable underrepresentation of Riptide sequencing data corresponding to GC-poor regions in *Brugia* and *Plasmodium* resulting in large tracts of low coverage in these genomes.

### No major differences in de novo assemblies in Riptide versus conventional Illumina

De novo assemblies of Riptide reads were less contiguous relative to non-Riptide counterparts. Yet, nearly all BUSCO genes were identified in *E. coli*, *Klebsiella*, and *Aspergillus* Riptide assemblies. BUSCO recovery was poor in the *Brugia* Riptide assembly, which may be a consequence of assembly fragmentation due to GC bias. A previous study demonstrated that strong GC bias in simulated Illumina reads decreases the contiguity of genome assemblies, however the fragmentation can be rescued with greater sequencing depth^24^. Nevertheless, there is an upper limit on assembly contiguity when using short read data, regardless of assembly method^25,26^. With the rapid adoption of long read sequencing^27^, small differences in assembly contiguity between Illumina-based libraries will likely be inconsequential. Rather, Riptide libraries provide a cost-effective solution for hybrid assemblies or short read data to be used for genome polishing.

### Direct estimate of contamination using Riptide libraries

Contamination can occur at many places in the sequencing pipeline, including traditional library preparation^28–30^. Multiple sources of potential contamination in our experiments include contamination inherent to the Riptide platform as well as pipetting errors and index misassignment during Illumina sequencing. Here, contamination could be directly assessed by (a) preparing libraries for diverse samples and subsequently mapping Riptide reads to a combined reference and (b) preparing libraries containing no gDNA as negative controls. Riptide reads predominantly mapped to their source genome and following data filtering procedures < 10% of all reads mapped to non-source genome sequences. A subset of nonspecific mappings are likely due to microhomology between sequenced organisms. However, additional filtering procedures may be needed in order to minimize well-to-well contaminant reads. This may be especially important for sequencing multiple individuals from a single species, where retention of contaminating sequences could lead to improper variant calling or misassemblies. Contaminants should be assessed regardless of library preparation method; non-Riptide libraries likely have similar contamination but it could not be assessed directly in the same manner.

### Effective sequencing of metagenomes

Riptide libraries are customizable with the use of low and high GC primer sets to amplify genomes of interest. Our sequencing of metagenomes illustrates the effect of primer selection on library composition. Increasing GC content of Riptide primers was associated with enrichment of reads assigned to high GC bacterial phyla, and there was a consistent association between primer selection and metagenome composition. Researchers using Riptide library preparations should use consistent primer sets when comparing compositions of metagenomes. Although there were observable differences between Riptide and NEBNext libraries, we are agnostic with respect to which library is more reflective of the actual biological diversity. A previous study has demonstrated that bacterial genome abundance estimates are prone to GC bias when using shotgun sequencing, and normalization procedures may be required for appropriate comparisons of metagenomes even when using a single set of primers in an experiment^31^.

### Cost-effective library preparation for high throughput sequencing experiments

A strong advantage of Riptide is the cost-effectiveness of library preparation: per-sample Riptide consumables is advertised at $10 versus $26 for KAPA Hyper and $22 for NEBNext Ultra^32–34^. Pooling Riptide libraries after the initial barcoding and polymerization steps represents a substantial reduction in library preparation time, reducing time costs of library preparation, thus reducing personnel costs. However, currently the only available kit contains materials for 960 reactions, and the total cost of this kit ($9600, July 2020) might be too large for some users.

## Conclusion

The Riptide kit offers notable cost reductions in library preparation for NGS experiments and will be particularly attractive to researchers with a need to sequence hundreds of samples at a time particularly to generate low-cost, high-fidelity short reads for error correction of long reads. Even so, Riptide is not an all-purpose replacement for Illumina library preparation, and some sequencing applications will benefit from additional modification of the Riptide protocol or alternate Illumina library preparations.

## Methods

### Biological samples

For *A. baumannii* str. 25493-3, *E. coli* str. HS, *K. pneumoniae* str. 4006 and *S. boydii* str. 5216-82, overnight cultures grown in L-broth were pelleted (12,000×*g*), resuspended in 50 mM Tris, 1 mM EDTA, 10 µl RNAse (20 mg/ml) and lysed with 0.4% SDS at 56 °C for 30 min. A 0.5 volume of 7.5 M ammonium acetate was added, and samples were incubated for 15 min on ice. Genomic DNA was extracted with phenol:chloroform:isoamyl alcohol followed by chloroform:isoamyl alcohol and precipitated with isopropanol. After two washes with 70% ethanol, the pellet was allowed to air dry and resuspended in water.

Plasmid pB171 genomic DNA was isolated by alkaline lysis^35^, subjected to a CsCl-ethidium bromide density gradient^36^, and dialyzed in milli-Q water (Millipore Sigma, Burlington, MA, USA).

*Aspergillus* str. 925, 928, H7810724, and T8015994 were prepared according as previously described^37^, with minor modifications. Briefly, the isolates were grown on potato dextrose agar plate at 30 °C for 5–7 days. Spores were collected in normal saline with 0.5% Tween 20. Approximately 10^8^ spores were inoculated in flask containing 100 ml minimal medium and incubated overnight with shaking at 30 °C. The mycelia were filtered using microcloth and washed with saline. Approximately 2 g mycelium was mixed with 1 ml lysis buffer (200 mM Tris–Cl, 0.5% SDS, 50 mM EDTA), then ground into powder under liquid nitrogen using a pre-cooled pestle and mortar. The ground mycelium was mixed with an equal volume of phenol/chloroform/isoamyl alcohol (25:24:1). The phases were separated by centrifugation at 13,000 rpm for 10 min at 4 °C. The aqueous layer was extracted with same volume of chloroform:isoamyl alcohol (24:1). DNA was precipitated from the aqueous layer with 2.5 volumes of 100% ethanol and 1/10 volume of sodium acetate (3 M, pH 5.2) at − 20 °C overnight. The DNA was recovered by centrifugation at 13,000 rpm for 10 min at 4 °C. The pellet was then washed with 70% ethanol, air-dried and resuspended in 500 µl TE buffer (10 mM Tris–HCl, 1 mM EDTA, pH 8.0). RNA was removed by incubating with 40 µl RNase A (10 mg/ml, Fisher Scientific, Hampton, NH, USA) at 37 °C for 15 min. DNA was purified with FastDNA spin kit (MP Biomedicals Inc., Irvine, CA, USA).

Genomic DNA from *Brugia pahangi* str. FR3 (NR-46542) was provided by the NIH/NIAID Filariasis Research Reagent Resource Center for distribution by BEI Resources, NIAID, NIH in TE buffer. Genomic DNA from *Cryptosporidium parvum* str. TU114 samples were obtained from purified oocysts in mouse fecal samples as previously described^38^.Genomic DNA was extracted from cultured *Plasmodium falciparum* str. NF54 parasites as previously described^39^. *Theileria parva* Muguga samples were obtained as previously described^40^. *T. parva* genomic DNA was produced using phenol/chloroform extraction from schizont-infected lymphocyte cell line cultures.

### Riptide library preparation and sequencing of isolates

Riptide libraries (iGenomX, Carlsbad, CA, USA) were prepared using the manufacturer’s protocol (v.1.03). In Riptide plate 1, the 96-well plate contained DNA from 89 biological samples alongside seven blank wells. In Riptide plate 2, samples included five additional *Acinetobacter* strains as well as 24 metagenomic samples. During the initial primer extension step, the GC primers used were based on genomic GC content of the biological sample (< 40% GC: low GC primer, 40–60% GC: low + high GC primer, > 60%: high GC primer). After sample pooling, DNA capture, and PCR steps, the library was size selected by adding 0.7 volumes of SPRI Beads II followed by 0.3 volumes of the same beads. The library quality was assessed on a Perkin Elmer GX with no cleanup and qPCR was used to quantify and dilute prior to loading for sequencing (2 × 150 bp) on a single lane using the Illumina HiSeq 4000 instrument (Illumina Inc., San Diego, CA, USA) and then demultiplexed. Riptide sample descriptions and SRA accessions are provided in Supplementary Data S1.

### Conventional Illumina library preparation and sequencing of isolates

In five of ten samples (Supplementary Data S1), gDNA extracted for Riptide library preparation was also used to prepare libraries using the KAPA Hyper Library Preparation Kit (Kapa Biosystems, Woburn, MA, USA). Four of five extracts (excluding *Shigella*) were sheared to 500 bp with a Covaris E220 instrument. Libraries were prepared using the manufacturer’s protocol, including a PCR amplification step, and size selected and quantified on a Perkin Elmer GX. Paired end libraries (2 × 150 bp) were sequenced on a single lane on an Illumina HiSeq 4000 (Illumina Inc., San Diego, CA, USA) and then demultiplexed. Sample information, including SRA accessions, are provided in Supplementary Data S1.

### Read representation

Read counts for each library were obtained from sequencing summary files provided by the Genomics Resource Center at the University of Maryland, Baltimore. Read counts were plotted for each study specimen, and the expected read count per Riptide library was estimated by dividing the total sequencing throughput of the Riptide run by the number of Riptide libraries containing gDNA (654/89 = 7.3 million reads). For seven samples that were consistently amplified, the read count population variance was calculated using the formula σ^2^ = (Σ(*X* − μ)^2^)/*N*, where *X* is the mean read count value, μ is the read count value from each replicate library, and *N* is the number of replicates. Variance values were plotted against corresponding genome sizes, and a linear regression with standard error was plotted in R.

### Riptide contamination analysis

Raw reads were mapped to a custom reference genome containing the combined genome sequences of all samples tested on the plate (Table 1) using bwa mem v.0.7.17^11^ with -k 23 and all other default parameters. Unmapped reads, duplicate reads, and non-primary alignments were removed from BAM files using SAMtools v.1.9^12^ and Picard v.2.5.0 (Broad Institute). After generating counts of reads mapping to each of the ten species, a custom script was used to visualize the proportion of total reads from Riptide libraries mapping to each genome. The same analyses were repeated following removal of non-primary alignments in addition to primary alignments with MAPQ < 20.

### Read mapping performance and genome coverage analysis

Raw reads from *E. coli* and *Klebsiella* replicate libraries in addition to merged *Aspergillus* and *Brugia* libraries were randomly subsampled with seqkit v.0.7.2^41^ by targeting a common depth sequenced by all Riptide and KAPA libraries (100X for *E. coli*, *Aspergillus*, *Brugia*; 75X for *Klebsiella*). Subsampled reads were aligned to their respective source genome sequences (Table 1) using bwa mem v.0.7.17 with -k 23 and all other default parameters. Primary alignments, unmapped reads, and duplicate reads were quantified using Picard MarkDuplicates. Percentages of primary alignments were quantified and visualized for 16 individual Riptide libraries and four KAPA libraries. A Welch’s t test was conducted to evaluate differences in primary alignments, unmapped reads, and duplicate reads when comparing Riptide libraries versus KAPA libraries.

For each BAM file, genome coverage data was collected with BEDTools v.2.15.0^42^. Using only primary alignments, sequencing depth data was collected using BEDTools ‘coverage’ with the -hist parameter. The observed sequencing depth distributions were compared against the expected depth mode, i.e. the targeted subsampling depth. To assess GC bias in sequencing depth, libraries were assessed using Picard CollectGcBiasMetrics. Reference genomes were divided into sliding windows using window size = 100 bp and step size = 0 bp. GC content was determined for each window, and the normalized sequencing depth was calculated as the average number of mapped reads per window at a particular GC percentage by the average number of mapped reads per window of all GC bins. Windows with normalized coverage > 2 were omitted from the analysis.

### De novo assembly

Subsampled reads from *E. coli*, *Klebsiella*, *Aspergillus*, and *Brugia* were de novo assembled using SPAdes v3.13.0^15^ using default parameters. Quantitative summaries for assembly contigs > 1 kbp were obtained with QUAST v.5.0.2^16^ using species-specific reference genome sequences and associated GFF files from NCBI (Table 1). The presence of highly-conserved genes was determined with BUSCO v.3.0.2^17,18^ using the bacteria or eukaryote OrthoDB v9 dataset^43^.

### Metagenome samples

Metagenomic DNA from human fecal samples were obtained in the study described in McArthur et al.^19^. The study was approved by the Institutional Review Boards of University of Maryland, Baltimore (UMB), the University of Massachusetts Medical School (UMMS) and the Office of Research Protections of the U.S. Army Medical Research and Materiel Command (since the cGMP production of the challenge inoculum and the clinical trial were supported by the Defense Advanced Research Projects Agency [DARPA] of the U.S. Department of Defense). All research was performed in accordance with the guidelines and regulations outlined in IRB. Written informed consent was obtained from healthy adult volunteers 18–49 years of age who were screened for the absence of chronic medical conditions, immunodeficiency, and a history of recent foreign travel to an endemic region or prior ETEC infection (natural infection or experimental challenge), or prior receipt of an ETEC vaccine. This source of cGMP H10407 challenge inoculum has been registered as an investigational new drug (BB-IND-15900) under the U.S. Food and Drug Administration.

Samples were preserved in RNALater (Ambion Inc., Austin, TX, USA) (equal weight/volume). Samples were vortexed, washed 3 times with phosphate buffered saline (PBS) pH 7.4 (Quality Biological Inc., Gaithersburg, MD, USA) and 500 µl was used for nucleic acid extraction. Cell lysis was achieved by first incubating samples with 5 µl lysozyme (10 mg/ml in 20 mM sodium acetate pH 4.5), 13 µl mutanolysin (11.7 U/ml), 3.2 µl lysostaphin (1 mg/ml in 20 mM sodium acetate pH 4.5) at 37 °C for 30 min followed by the addition of 10 µl proteinase K (20 µg/ml), 50 µl 10% SDS, 2 µl RNAse A (10 mg/ml) and incubating 55 °C for 45 min. The samples were transferred to a FastPrep Lysing Matrix B tube (Bio101) and microbial cells were lysed by mechanical disruption using a FastPrep bead beater (MP Biomedicals Inc., Irvine, CA, USA) set at 6.0 m/s for 30 s. The lysate was further processed using the Quick-DNA Fecal/Soil Microbe Microprep kit (Zymo Research, Irvine, CA, USA) according to manufacturer’s instructions, picking up from the transfer step of the supernatant to the Zymo-Spin III-F filter. Unless otherwise specified, all reagents were purchased from Sigma-Aldrich (St. Louis, MO, USA).

### Riptide metagenome library preparation and sequencing

Riptide libraries were prepared for four metagenomic samples representing four time points of the ETEC challenge experiment (day 1, day 2, day 6, day 10). For each sample, Riptide libraries were prepared using three primer sets (low GC, high GC, 1:1 low:high GC) and two technical replicates were generated for each sample-primer combination. Samples were pooled and purified with SPRI Beads II prior to sequencing (2 × 150 bp) on an Illumina HiSeq 4000 instrument (Illumina Inc., San Diego, CA, USA) followed by demultiplexing.

### Conventional metagenome library preparation and sequencing

NEBNext Ultra DNA libraries were generated for each metagenome sample following the manufacturer’s protocol. Genomic DNA was sheared with a Covaris instrument and either 100 ng (Day 6) or 500 ng (Day 1; Day 2; Day 10) DNA was used in library preparations. Libraries were size-selected with SPRI Beads II and amplified with PCR for 10 cycles. Libraries were sequenced on the Illumina HiSeq2500.

### Metagenome analysis

Raw read counts were obtained for each metagenomic Riptide library from sequencing summary files provided by the Genomics Resource Center at the University of Maryland, Baltimore. Taxonomic assignment of reads was conducted using kraken2 v.2.0.8-beta^19^. Briefly, kraken2 uses a k-mer matching system to assign each sequence to the lowest common ancestor (LCA) present in a reference database. Here, reads were assigned to LCA based on the NCBI NT database (downloaded 2017-04-05). The output from kraken2 was parsed to retrieve the total number of sequences with a taxonomic assignment, the percentages of reads with various domain classifications (i.e. archaea, bacteria, eukaryote, viruses), and the percentages of reads with various phylum classifications. At the phylum level, LCA percentages were only reported for groups with > 0.5% of total reads. Read sets from high and low primer GC were combined to mimic the replicates sequenced with both primers in the 1:1 ratio.

### Ethics approval and consent to participate

Metagenomic DNA from human fecal samples were obtained in the study described in McArthur et al.^19^ with written informed consent. The design of this work was approved by the Institutional Review Boards at the University of Maryland Baltimore (HP-00059433).

## Supplementary Information

Supplementary Information 1.

Supplementary Information 2.

## Data Availability

All the data supporting the conclusions of this article have been deposited in Genbank/EMBL/DDBJ Sequence Read Archive under BioProjects PRJNA575185, PRJNA338184, PRJNA194878, PRJNA437480, PRJNA577466, and PRJNA312679. SRA accessions for individual libraries are available in Supplementary Data S1. All commands and scripts used in the study are available at https://github.com/Dunning-Hotopp-Lab/Riptide_analysis.
