# Supplementary material for: Evaluation of a high-throughput, cost-effective Illumina library preparation kit
Source: Sci Rep. 2021 Aug 5;11:15925. doi: 10.1038/s41598-021-94911-0 (PMC8342411; doi:10.1038/s41598-021-94911-0)
Supplement: Supplementary file 2 — Supplementary Information 2. [file 41598_2021_94911_MOESM2_ESM.pdf]

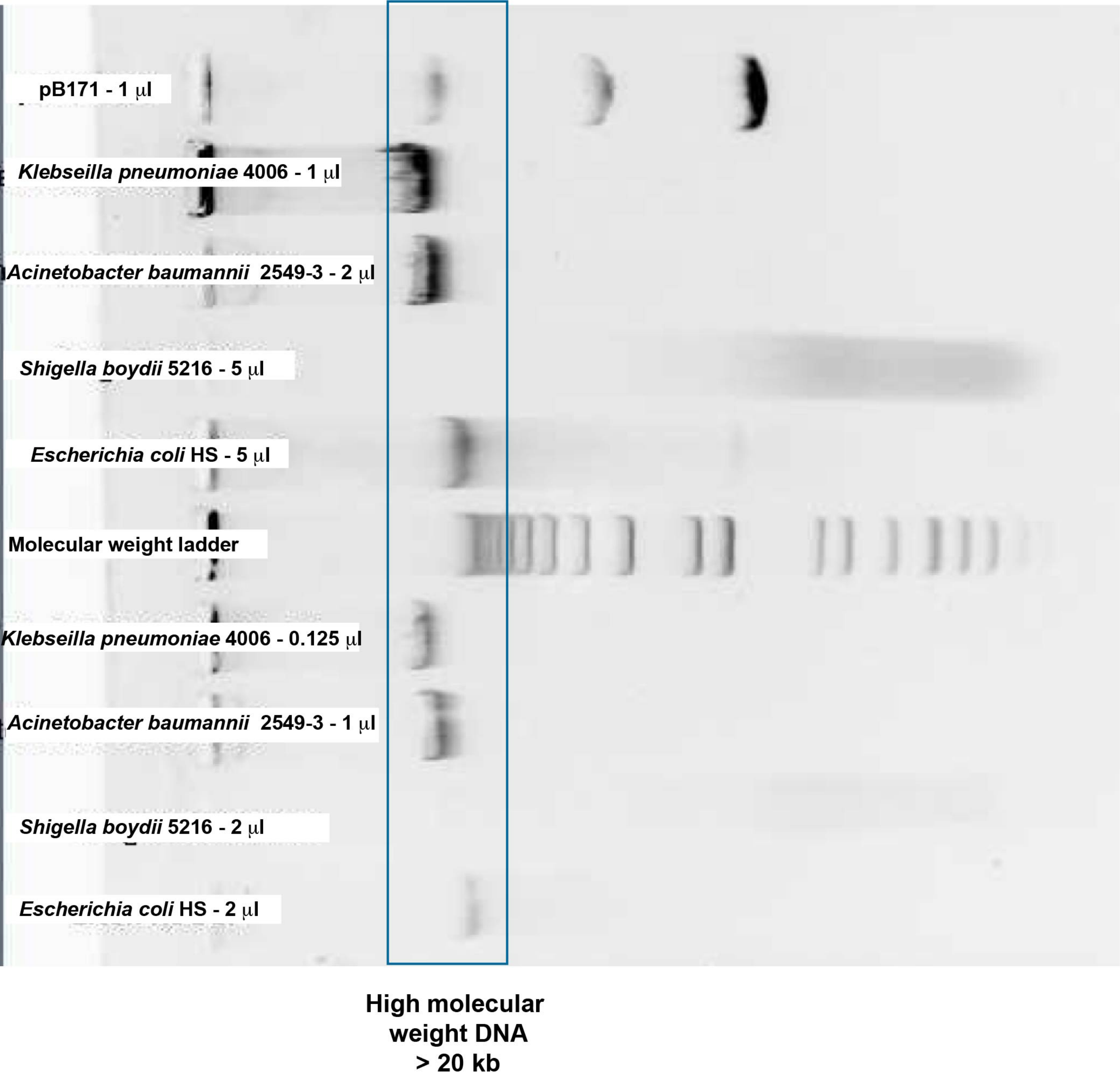

Supplementary Figure S1. Gel electrophoresis of prokaryotic gDNA used in Riptide and KAPA library preparations

Agarose gel electrophoresis was used to verify composition of five prokaryotic gDNA samples used to construct Riptide libraries. Lanes are labeled by sample. The visible bands in the blue box indicate the presence of high molecular weight DNA in four of five samples, whereas there was no distinct band in the degraded *Shigella* gDNA.

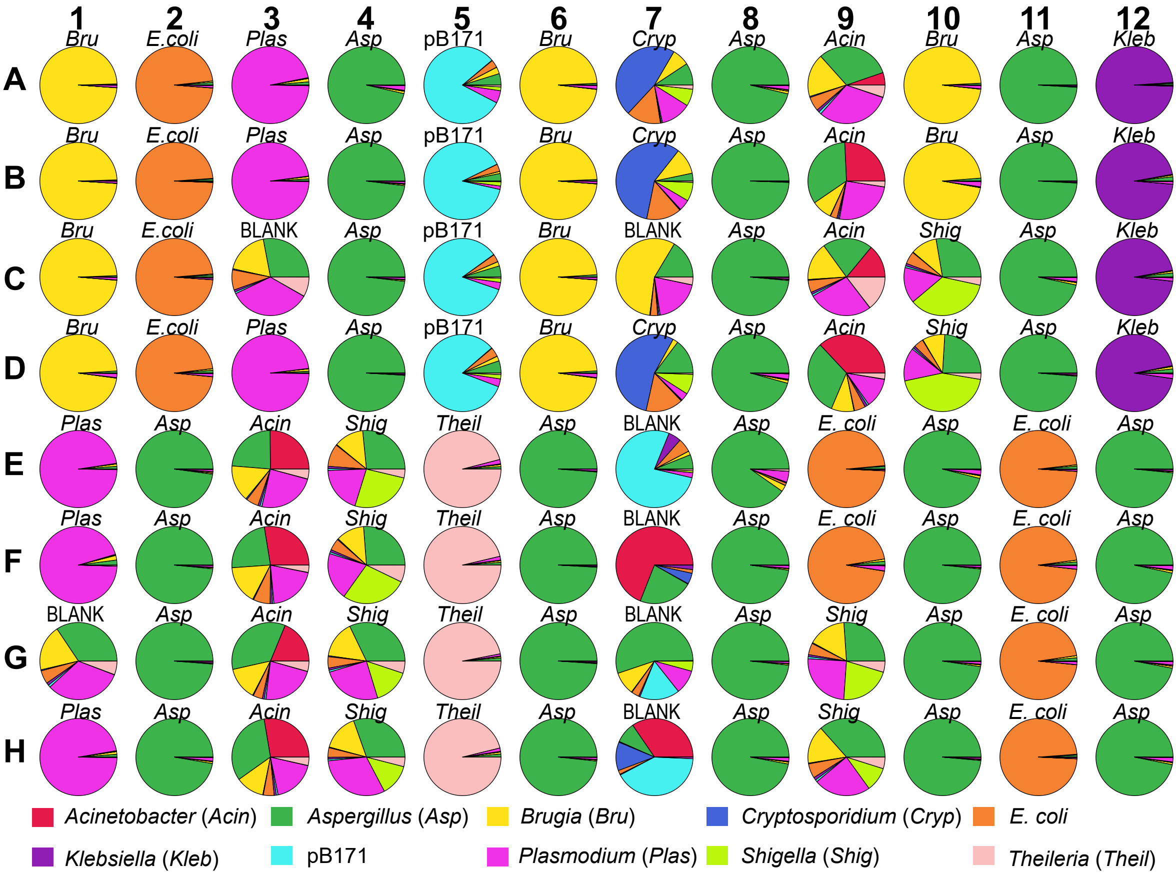

Supplementary Figure S2. Contamination assessment of Riptide samples after removal of MAPQ <20 reads

For each Riptide library, primary reads were mapped to a reference containing whole genomes from all specimens used in the study. Reads with MAPQ <20 were subsequently removed from each BAM file. Pie charts display proportions of reads mapping to each genome. The figure is organized to represent the actual position of samples loaded on the Riptide 96-well plate. Wells are labeled according to source DNA used to prepare the Riptide libraries; wells containing no gDNA are labeled as BLANK.

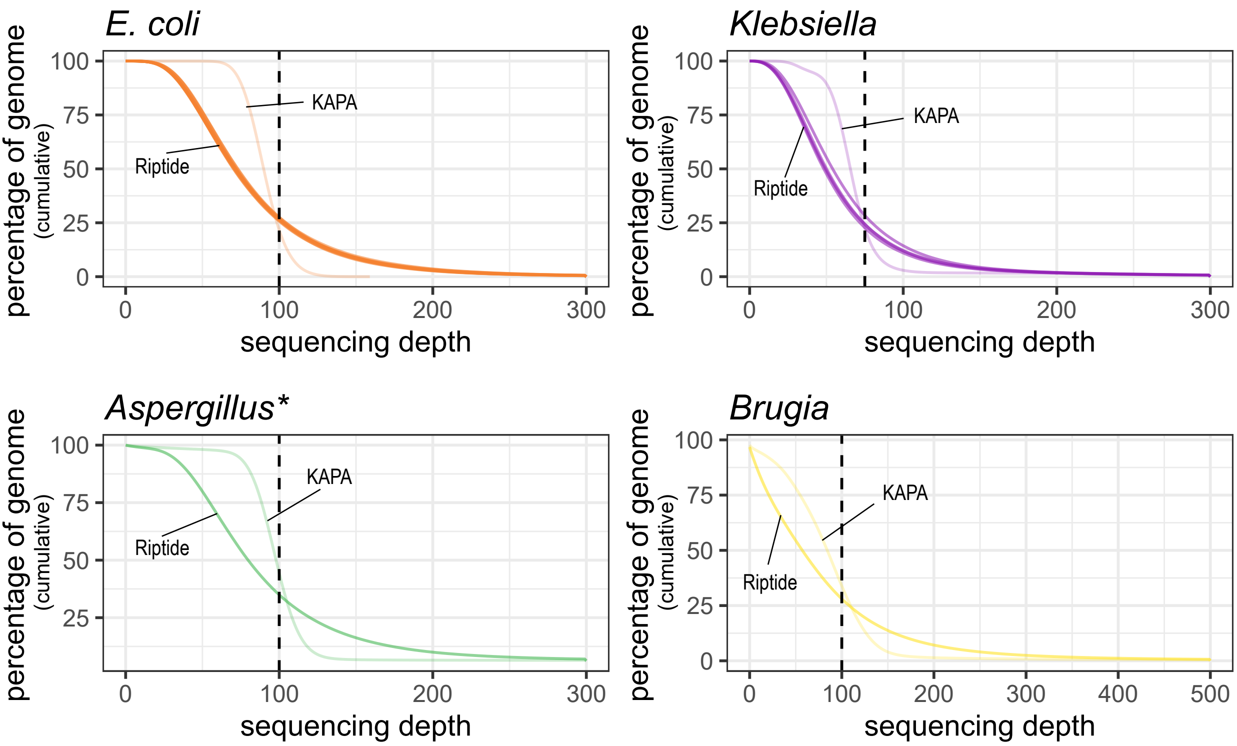

Supplementary Figure S3. Cumulative percentage histograms of sequencing depth in Riptide and non-Riptide libraries

For each library, randomly subsampled reads were mapped to a reference containing the source genome, and non-primary alignments were subsequently removed. All Riptide replicates are shown for *E. coli* and *Klebsiella*, while distributions for *Aspergillus* and *Brugia* were generated from merged Riptide libraries. Vertical dotted lines correspond to the targeted sequencing depth with subsampling procedures. *Regions having zero sequencing depth which largely represent tracts of Ns in the scaffolded *Aspergillus* assembly were omitted from this plot.

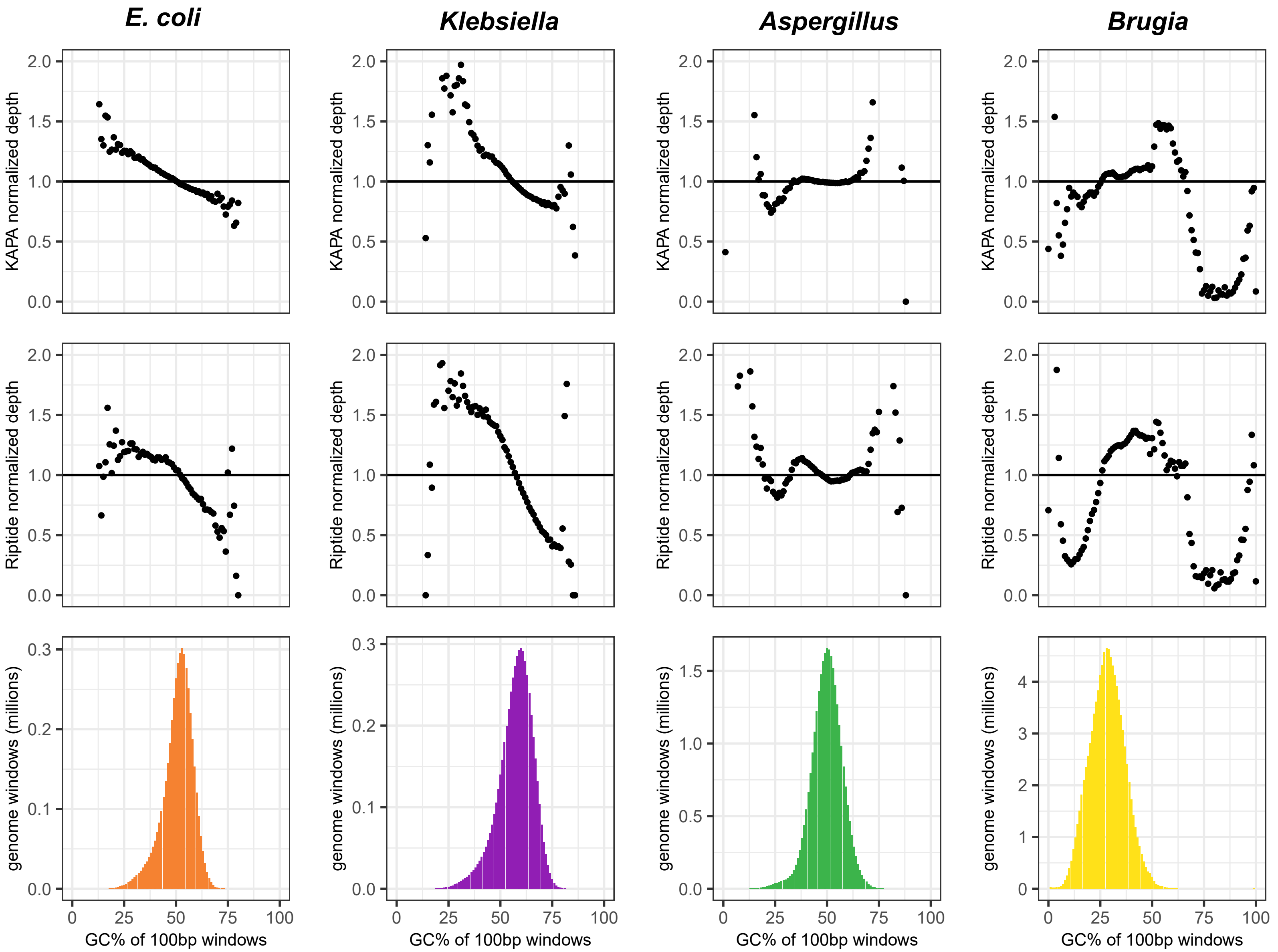

Supplementary Figure S4. Assessment of GC bias in Illumina libraries

A sliding window analysis was performed to calculate GC content and sequencing depth statistics using Picard CollectGcBiasMetrics with window size = 100 bp and step size = 0 bp. Bottom: histograms represent counts of windows corresponding to each GC percentage value. Middle: normalized sequencing depth was calculated for Riptide libraries by dividing the average number of mapped reads per window at a particular GC percentage by the average number of mapped reads per window of all GC bins. Normalized depth for *E. coli* and *Klebsiella* are shown for representative replicates. Normalized depth for *Aspergillus* and *Brugia* are shown for merged Riptide replicates. Top: normalized sequencing depth was calculated for KAPA libraries. Normalized depth values >2 were omitted from the analysis.
